# Identification of 2,4‐Dinitro‐Biphenyl‐Based Compounds as MAPEG Inhibitors

**DOI:** 10.1002/cmdc.202200327

**Published:** 2022-10-11

**Authors:** Simone Di Micco, Stefania Terracciano, Martina Pierri, Vincenza Cantone, Stefanie Liening, Stefanie König, Ulrike Garscha, Robert Klaus Hofstetter, Andreas Koeberle, Oliver Werz, Ines Bruno, Giuseppe Bifulco

**Affiliations:** ^1^ European Biomedical Research Institute of Salerno (EBRIS) Via Salvatore De Renzi 50 84125 Salerno Italy; ^2^ Department of Pharmacy University of Salerno Via Giovanni Paolo II 132 84084 Fisciano SA Italy; ^3^ Department of Pharmaceutical/Medicinal Chemistry Institute of Pharmacy Friedrich-Schiller-University Jena Philosophenweg 14 7743 Jena Germany; ^4^ Michael Popp Research Institute University of Innsbruck Mitterweg 24 6020 Innsbruck Austria

**Keywords:** LTC_4_S inhibitors, FLAP inhibitors, multitargeting approach, anti-inflammatory drugs, anticancer agents

## Abstract

We identified 2,4‐dinitro‐biphenyl‐based compounds as new inhibitors of leukotriene C_4_ synthase (LTC_4_S) and 5‐lipoxygenase‐activating protein (FLAP), both members of the “Membrane Associated Proteins in Eicosanoid and Glutathione metabolism” (MAPEG) family involved in the biosynthesis of pro‐inflammatory eicosanoids. By molecular docking we evaluated the putative binding against the targets of interest, and by applying cell‐free and cell‐based assays we assessed the inhibition of LTC_4_S and FLAP by the small molecules at low micromolar concentrations. The present results integrate the previously observed inhibitory profile of the tested compounds against another MAPEG member, i. e., microsomal prostaglandin E_2_ synthase (mPGES)‐1, suggesting that the 2,4‐dinitro‐biphenyl scaffold is a suitable molecular platform for a multitargeting approach to modulate pro‐inflammatory mediators in inflammation and cancer treatment.

## Graphical abstract

**Multipronged strategy**: 2,4‐Dinitro‐biphenyl‐based compounds were identified as inhibitors of leukotriene C_4_ synthase (LTC_4_S) and 5‐lipoxygenase‐activating protein (FLAP), both members of the “Membrane Associated Proteins in Eicosanoid and Glutathione metabolism” (MAPEG) family involved in the biosynthesis of pro‐inflammatory eicosanoids. The results of this study show the 2,4‐dinitro‐biphenyl scaffold to be suitable for a multitarget approach to modulate pro‐inflammatory mediators in inflammation and cancer treatment.

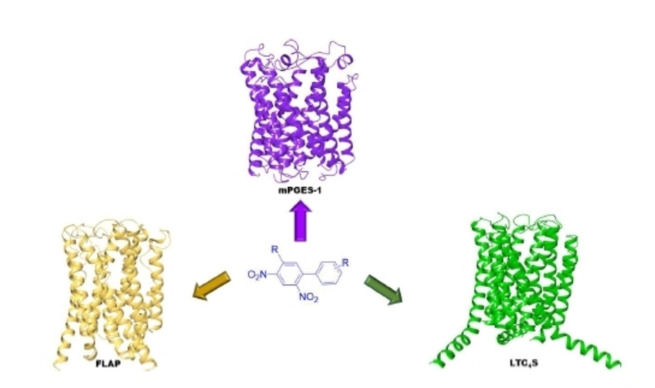

## Introduction

Eicosanoids, including prostaglandins (PGs) and leukotrienes (LTs), are a family of potent lipid mediators involved in many physiological processes. However, increased biosynthesis of pro‐inflammatory eicosanoids has been implicated in diverse chronic inflammatory pathologies like rheumatoid arthritis and cancer. Eicosanoids are generated via oxygenation of arachidonic acid along cyclooxygenase (COX) and lipoxygenase (LOX) pathways. The Membrane Associated Proteins in Eicosanoid and Glutathione metabolism (MAPEG) family comprises LTC_4_ synthase (LTC_4_S), 5‐lipoxygenase‐activating protein (FLAP) and microsomal prostaglandin E_2_ synthase (mPGES)‐1, all sharing a high sequence and structure homology.^[1]^ Indeed, different mPGES‐1 inhibitors, such as MK886^[2]^ or LAF9,^[3]^ block the activity of both FLAP and LTC_4_S. The latter catalyses the production of LTC_4_ from LTA_4_, whereas FLAP is necessary for the activation of 5‐lipoxygenase (5‐LOX) in intact cells to produce LTs, 5‐hydroxyeicosatetraenoic acid, 5‐oxo‐eicosatetraenoic acid, and specialized pro‐resolving mediators (SPM) of the lipoxin and resolvin classes. MAPEGs are considered promising drug targets,[^4^, ^5^] indeed, mPGES‐1 inhibitors are proposed as alternatives to NSAIDs.[^6^, ^7^] The latters, by suppressing PGs formation through COX‐1/2 inhibition, increase LT production giving rise to side effects.^[8]^ In this context, multitargeting strategies[^9^, ^10^] for concurrent inhibition of PGE_2_ and LT biosynthesis, based on inhibitors[^11^, ^12^] blocking related proteins, are particularly pursued for development of safer therapeutical treatment of inflammation and cancer. Recently, we identified potent 1‐fluoro‐2,4‐dinitro‐biphenyl‐based mPGES‐1 inhibitors which did not affect COXs activities.^[13]^ These interesting results prompted us to investigate our 1‐fluoro‐2,4‐dinitro‐biphenyl lead compounds as binders of other MAPEG members, LTC_4_S and FLAP, for multitarget drug development, by combining molecular docking and biological assays.

## Results and Discussion

**Figure 1:**
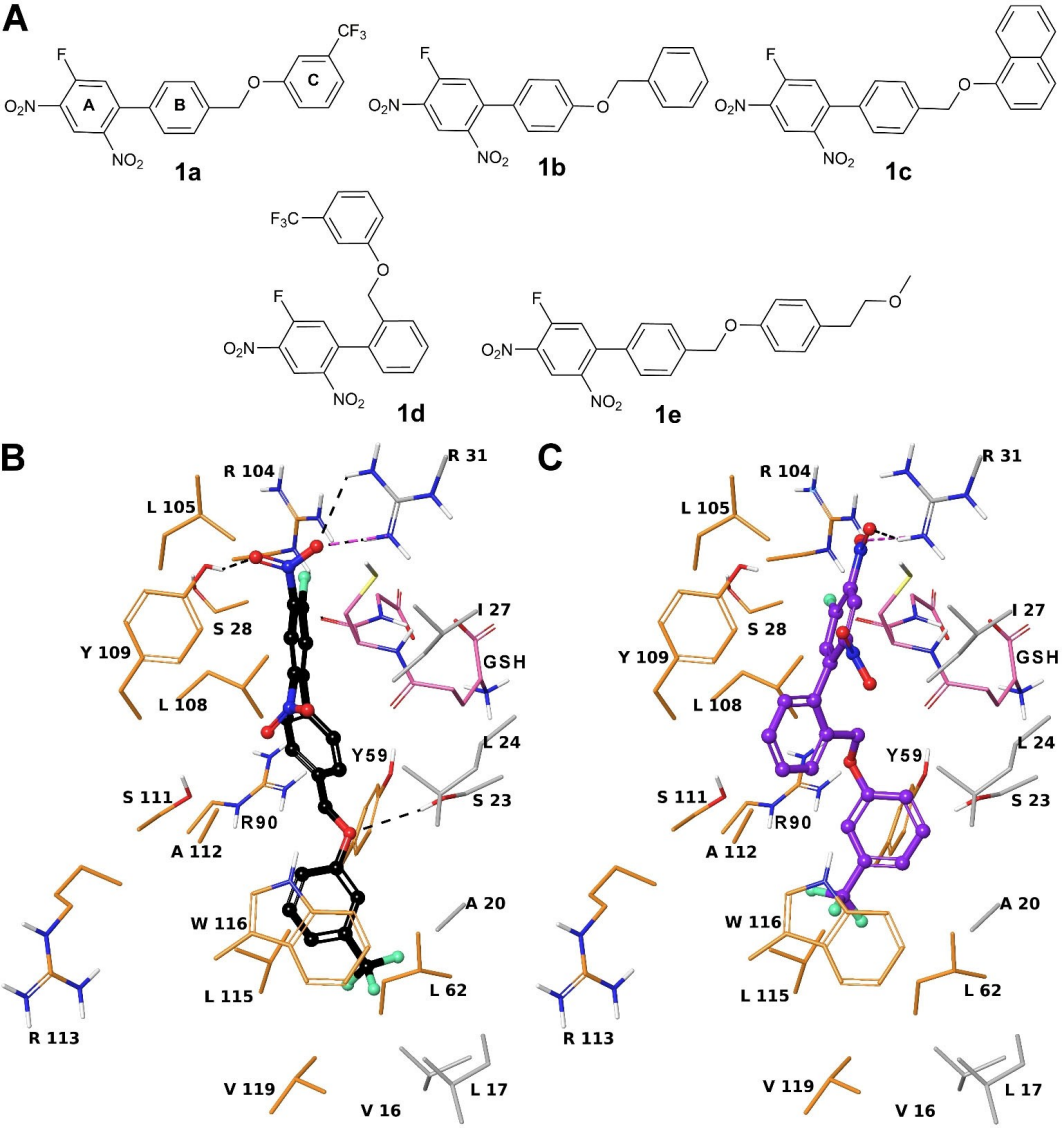
(A) **1 a**‐**e** chemical structures. Three‐dimensional model of the interactions given by **1 a** (B) and **1 d** (C) with LTC_4_S. The protein is represented by orange (chain A) and grey (chain C) tube (C, according to chain colour; polar H, white; N, dark‐blue; O, red; S, yellow), whereas the GSH by faded‐salmon tube with the same atom colour code of the enzyme, except for C (faded‐salmon). The ligands are depicted by sticks (**1 a**, black; **1 d**, violet) and balls (C, as for the sticks; polar H, white; N, dark‐blue; O, red; F, light green). The dashed black and violet lines indicate the H‐bonds and salt bridges, respectively.

We performed an *in silico* screening of our mPGES‐1 inhibitors (**1a**‐**e**, Figure 1A) to evaluate putative binding towards two valuable protein targets converging for useful multitargeting approach against biosynthesis of pro‐inflammatory eicosanoids: LTC_4_S and FLAP. Similarly to our developed inverse virtual screening protocol,[^14^, ^15^] we compared the docking scores of **1 a**‐**e** with the values obtained for a set of 250 generated decoys, followed by visual inspection to detect plausible poses. Heterogeneous docking results were obtained for **1 a**‐**e** against LTC_4_S. From the comparison with decoys (see computational details), **1 a** and **1 d** stood out, whereas **1 c** and **1 e** showed lower docking scores than averaged values of decoys. Indeed, compounds **1 c** and **1 e** do not optimally accommodate in the enzymatic pocket, mainly due to the larger size of ring C moiety.^[16]^ A better positioning was observed for **1 a** and **1 d**. Indeed, the latter molecules fit well the hydrophobic crevice formed by Trp116, functional for a proper positioning of lipophilic substrate.^[16]^ The docked pose of **1 a** presents the nitro group at C‐2 engaged in H‐bond with Arg31 and Tyr109 (Figure 1B). The ring A of **1 a** gives π‐π interactions with Tyr109 and van der Waals contacts with Ile27 (Figure 1B). The ring B forms van der Waals interactions with Leu24, Ile27, Ile108, Tyr109, Ala112. The ring C is involved in π‐π interaction with Trp116, aromatic H‐bond with the backbone CO of Ala112 and van der Waals contacts with Ala112, Leu115, Val119, Tyr59, Leu17, Ala20, Leu62 (Figure 1B). The oxymethylene is H‐bonded to Ser23. Compared to **1 a**, the congener **1 b** does not give H‐bonds with Ser23, Tyr109 and Ala112, and it also lacks van der Waals interaction with Tyr109, as also showed by comparison with decoys. These lower numbers of interactions given by **1 b** suggested a lower/absent biological activity with respect to **1a** and **1d** as experimentally verified (see below). For **1 d**, we observed that both nitro groups at C‐2 and C‐4 to be salt‐bridged with Arg104 and Arg31, respectively (Figure 1C). Its ring B forms T‐shaped π–π interactions with Tyr109 and Trp116, whereas ring C gives the same interaction with Tyr59 and an aromatic H‐bond with CO of Ala20 (Figure 1C).

**Figure 2:**
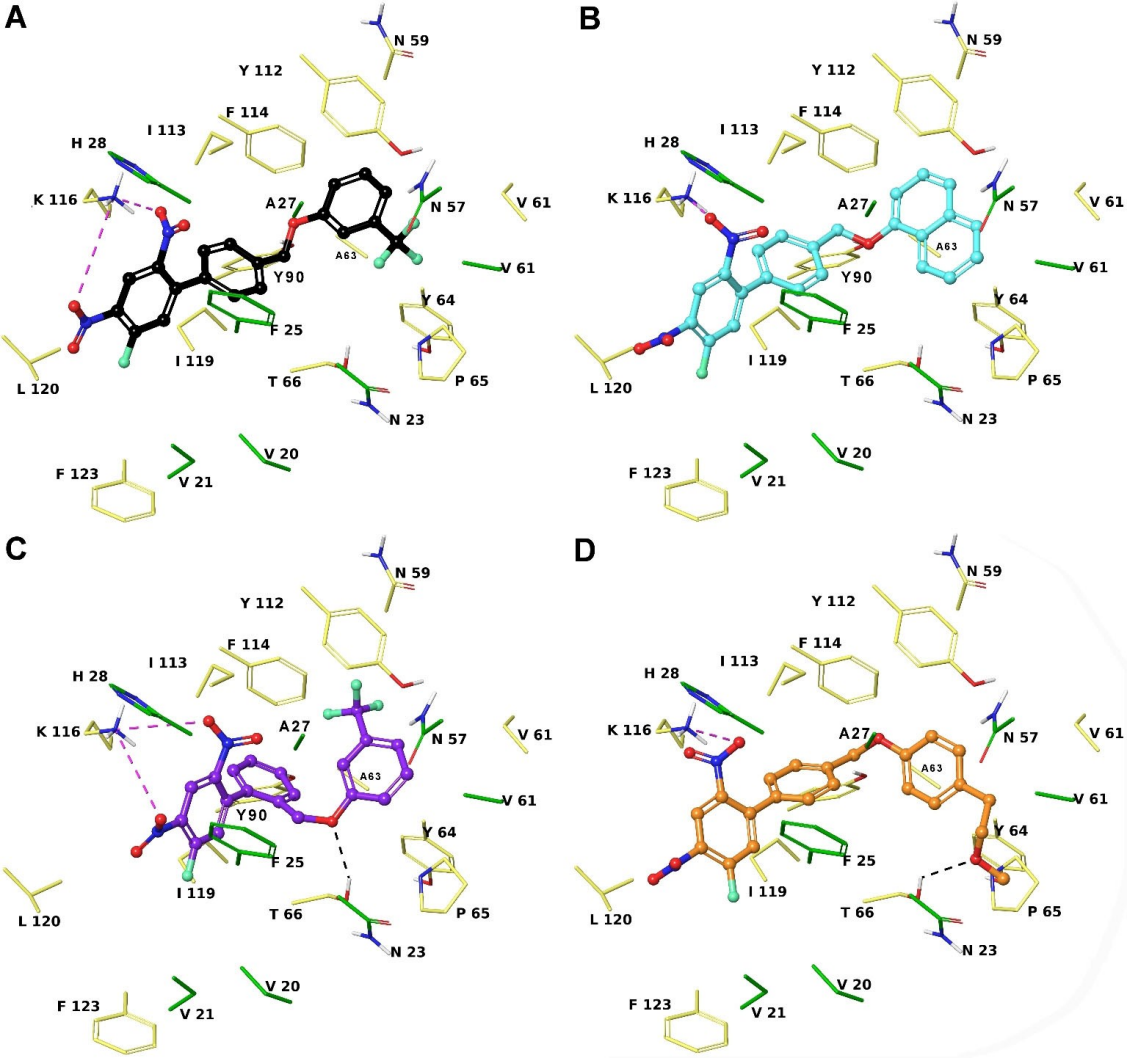
Three‐dimensional model of the interactions given by **1 a** (A), **1 c** (B), **1 d** (C) and **1 e** (D) with FLAP. The protein is depicted by faded‐yellow (chain A) and green (chain C) tube (C, according to chain colour; polar H, white; N, dark‐blue; O, red). The ligands are represented by sticks (**1 a**, black; **1 c**, cyan; **1 d**, violet; **1 e**, orange) and balls (C, as for the sticks; polar H, white; N, dark‐blue; O, red; F, light green). The dashed black and violet lines indicate the H‐bonds and salt bridges, respectively.

Concerning the screening against FLAP, **1 a**‐**e** showed a better docking score profile with respect to decoys, with **1 c** and **1 d** resulting top‐ranked. The compound **1 b** presented the lowest affinity profile compared to its congeners, indeed, it accounts for a lower number of interactions. Similar binding poses were observed for **1 a** and **1 c**‐**e** (Figure 2). In particular, the ring C deeply accommodates into the inner part of the binding pocket,^[17]^ establishing van der Waals contacts with: Asn23, Val61, Asn62, Ala63, Tyr112, Phe114. For **1 a** and **1 c‐e**, we also found an aromatic H‐bond by ring C with CO backbone of Tyr112. **1 d** and **1 e** also give the same interaction with CO of Asn59 and side chain of Thr66, respectively (Figure 2). The docked poses of **1 d** and **1 e**, respectively, show the oxymethylene and 2‐methoxyethyl groups to be involved in an H‐bond with the side chain of the Thr66. The ring B of **1 a‐c** and **1 e** gives van der Waals contacts with Gly24, Ala27, Ile113, Lys116, Ile119. The ring B, presenting the ring C in *ortho* position, establishes van der Waals contacts with Thr66 and Ile119. In addition, the ring A establishes a T‐shaped π‐stacking with Phe123. The nitro group at C‐4 of **1 a**‐**e** establishes an ionic interaction with Lys116, while the nitro group at C‐2 is involved in a π‐cation interaction with Phe123 side chain. Finally, the ring A of **1 a** and **1 c‐e** forms van der Waals contacts with His28 and Ile116, whereas for **1 a** the same moiety interacts with Val20, Val21, Lys116 and Leu120. Interestingly, the interactions given by rings A−C are observed for previously described co‐crystallized compounds with FLAP,^[17]^ and are essential to assure the intermolecular recognition.

**Figure 3:**
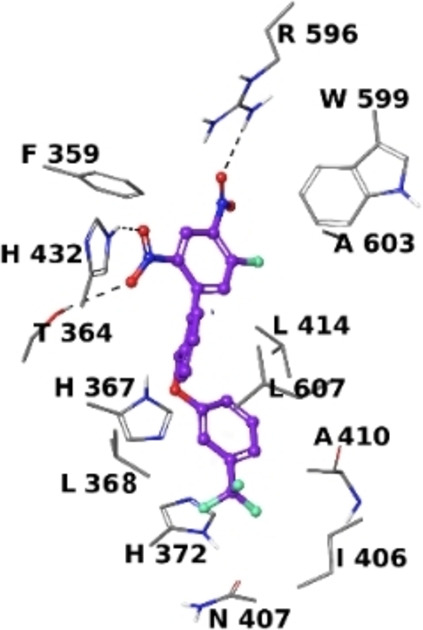
Three‐dimensional model of interactions given by **1 d** with 5‐LOX. The protein is depicted by grey tube (C, grey; polar H, white; N, dark‐blue; O, red). The ligand is represented by violet sticks and balls (C, as for the sticks; polar H, white; N, dark‐blue; O, red; F, light green). The dashed black lines indicate the H‐bonds.

Incidentally, our experimental investigations revealed the 5‐LOX as potential target for **1 d**, whose binding pose was deeply analysed (Figure 3). We found that the nitro group at C‐2 interacts with Arg596, whereas the C‐4 nitro groups is H‐bonded to Thr364 and His432. The ring A gives contacts with Phe359 and Trp599, whereas the ring B establishes van der Waals interactions with Leu368, Leu414 and Leu607. Finally, the ring C is involved in π‐π interactions with His367 and His372, and in van der Waals contacts with Ile406, Asn407 and Ala410.

**Figure 4:**
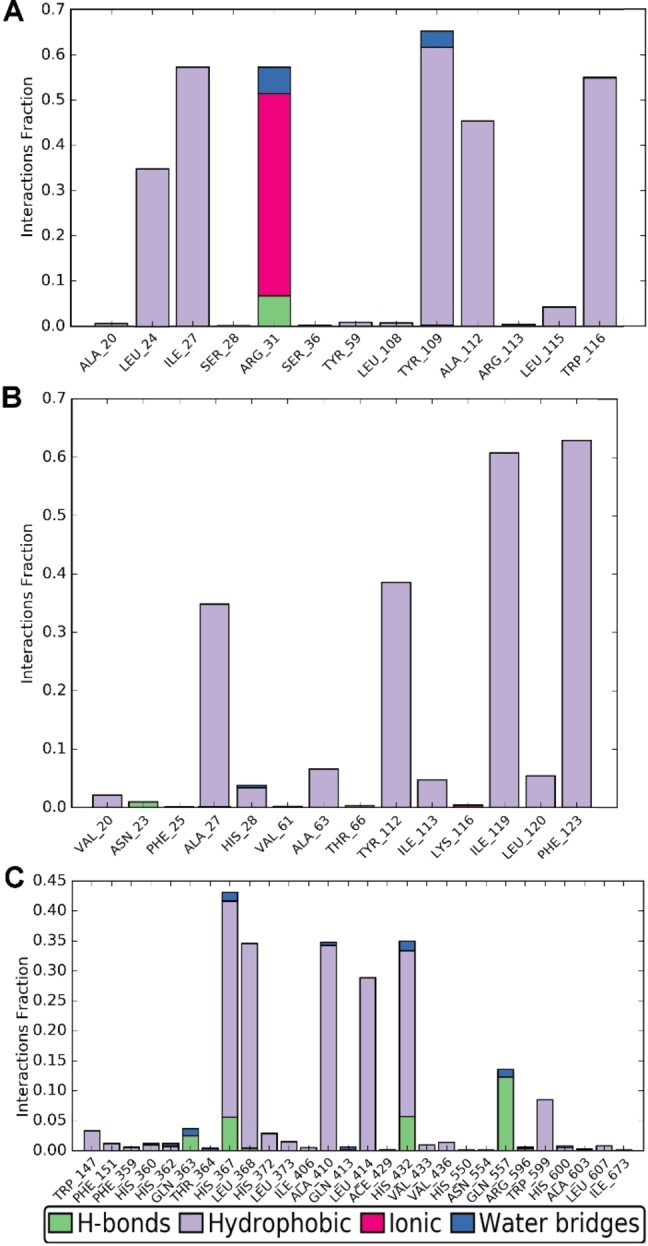
Contact histograms during the simulation of **1 d** with LTC_4_S (A), FLAP (B) and 5‐LOX (C).

In order to evaluate the time stability of the key interactions found by molecular docking investigation of **1 d** against LTC_4_S, FLAP and 5‐LOX, we carried out molecular dynamics simulations (100 ns, 310 K).[^18^, ^19^] For each investigated trajectory, most of the contacts with macromolecular residues (Figure 4), observed from the docked poses of **1 d**, were maintained during the whole simulation (>30 %), confirming their important contribution to complex stability.

**Figure 5:**
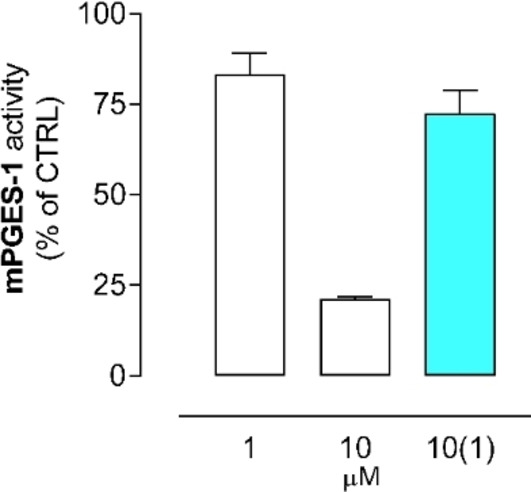
Catalytic activity of mPGES‐1 in presence of two **1 d** concentrations (1 and 10 μM) and after 10‐fold dilution from 10 to 1 μM (=10(1)). The cyan bar indicates the washout salvaged enzymatic activity.

Collectively, the theoretical screening suggested that the 1‐fluoro‐2,4‐dinitro‐biphenyl‐based scaffold was endowed with a potential property to bind also LTC_4_S and FLAP, besides mPGES‐1. As fluorinated electron‐poor aromatic rings are employed as covalent warheads binding cysteine thiols,^[20]^ we investigated the possible covalent bond formation between **1 d** and GSH by washout experiment with mPGES‐1. We experimentally observed that **1 d**, at concentrations of 1 and 10 μM, reduced the residual enzyme activity to 83.3 % and 21.3 %, respectively (Figure 5). Washout (10‐fold dilution) salvaged enzymatic activity (72.5 %), indicating a reversible mechanism of inhibition. Moreover, no adducts of **1 d** with GSH upon incubation of the both were detected by UPLC‐MS/MS in either Q1 scan or information‐dependent acquisition of enhanced product ions (EPI) (data not shown). The experiments were in agreement with the observed averaged distance from docked complexes between GSH thiol and C‐1 of ligands: 4.62 Å for mPGES‐1 and 4.92 Å for LTC_4_S.

Thus, compounds **1 a**‐**e** were synthesized as previously reported,^[13]^ and were then tested for their inhibition of LTC_4_S and FLAP. Concerning LTC_4_S enzyme, the compounds were tested in microsomal preparations of HEK293 cells, stably expressing human recombinant LTC_4_S. Among them, **1 a** and **1 d** significantly inhibited LTC_4_S activity (Table 1). On the contrary, compounds **1 b**, **1 c** and **1 e** did not show inhibitory activity towards the LTC_4_S enzyme (Table 1).

**Table 1:** Docking scores, V values, inhibition of mPGES‐1, LTC_4_S and 5‐LOX (given as IC_50_ values) in cell‐free enzyme assays, and of FLAP‐dependent 5‐LOX product formation in intact cells by compounds **1 a**‐**e**.

					IC_50_ [μM]			
	LTC_4_S		5‐LOX		cell‐free			cell‐based
compound	docking score (kcal/mol)	V	docking score (kcal/mol)	V	mPGES‐1	LTC_4_S	5‐LOX	5‐LOX
**1 a**	−8.915	1.531	‐8.238	1.350	0.26±0.9^[a]^	1.68±0.21	n.d.^[b]^	3.93±0.24
**1 b**	−7.559	1.298	‐7.451	1.221	0.60±1.2^[a]^	>100	n.d.	>100
**1 c**	−5.284	0.908	‐10.167	1.666	0.18±1.1^[a]^	>100	n.d.	4.18±0.16
**1 d**	−8.113	1.394	‐10.259	1.681	0.54±1.3^[a]^	0.93±0.04	3.35±0.22	1.40±0.09
**1 e**	−4.789	0.823	‐8.337	1.366	1.64±1.7^[a]^	>100	n.d.	3.31±0.27

[a] Reported in the literature.^[13]^ [b] n.d.=not determined.

**Figure 6:**
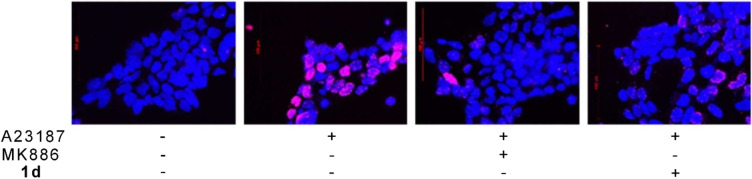
Effect of **1d** on 5‐LOX/FLAP interaction in HEK293 cells stably expressing 5‐LOX and FLAP. Cells were pre‐incubated with **1 d** (3 μM), MK886 (0.3 μM) or vehicle (0.1 % DMSO) for 10 min at 37 °C and afterwards stimulated by 2.5 μM Ca^2+^‐ionophore A23187 for 10 min at 37 °C. DAPI (blue) was used to stain the nucleus and proximity ligation assay (PLA) signals (magenta dots) visualize in situ 5‐LOX/FLAP interactions. Results are representative for 100 individual cells analyzed in three independent experiments.

Due to the lack of a cell‐free assay for studying the inhibition of FLAP that displays no enzyme activity, we used indirect analysis of 5‐LOX product formation in intact cells, which depends on the presence and functionality of FLAP, to evaluate the functional interference of our compounds with FLAP. In detail, the compounds were tested for their putative inhibitory activities against 5‐LOX in human neutrophils stimulated with 2.5 μM Ca^2+^ ionophore A23187. All compounds, except **1 b**, potently inhibited the formation of LTB_4_, its trans isomers and 5‐H(p)ETE (Table 1). Notably, such suppression of 5‐LOX product formation in intact cells could also be ascribed to direct inhibition of 5‐LOX enzyme besides or in addition to interference with FLAP.^[21]^ Therefore, we investigated on one hand if compounds **1 a‐e** could directly inhibit 5‐LOX activity independent of FLAP by using a cell‐free assay,[^22^, ^23^] and, on the other hand, if they disrupt the functional 5‐LOX/FLAP interaction by interference with FLAP using a proximity ligation assay.^[24]^ The compounds were tested for the inhibitory activities against human recombinant 5‐LOX enzyme in cell‐free assay using 20 μM arachidonic acid as substrate.[^22^, ^23^] None of the compounds, except for **1 d** (Table 1), inhibited 5‐LOX activity (Table 1). Therefore, the comparison of the outcomes from intact cells versus cell‐free assays suggest that, in intact cells, the compounds target FLAP and thus prevent 5‐LOX product formation.^[21]^ Moreover, we tested if **1 d**, the most active compound, would prevent the 5‐LOX/FLAP complex assembly using the proximity ligation assay.^[24]^ The assay was conducted in HEK293 cells, stably transfected with human recombinant 5‐LOX and FLAP, stimulated with 2.5 μM Ca^2+^ ionophore A23187, where 5‐LOX and FLAP interact with each other and FLAP inhibitors can prevent this interaction.[^21^, ^24^] As reference compounds we used the well‐known FLAP inhibitor MK886 and the 5‐LOX inhibitor zileuton, because we have already reported the inefficiency of zileuton in this assay with HEK cells under the same experimental conditions, while MK886 does block the interaction under these conditions.[^21^, ^25^] In analogy to the well‐recognized FLAP inhibitor MK886, **1 d** was able to block the 5‐LOX/FLAP interaction (Figure 6), confirming our assumption that its activity on cellular 5‐LOX product formation is due to the interference with FLAP, besides direct inhibition of 5‐LOX.

## Conclusion

We demonstrated that our previously identified 1‐fluoro‐2,4‐dinitro‐biphenyl‐based mPGES‐1 inhibitors also interfere with two structurally related MAPEGs, namely LTC_4_S and FLAP. Compounds **1 a** and **1 d** inhibit LTC_4_S in the low micromolar range, showing comparable inhibition efficiency as against mPGES‐1. Interestingly, compounds **1 a** and **1 c**‐**e** were also able to inhibit cellular 5‐LOX product biosynthesis (LTB_4_, its *trans* isomers and 5‐H(p)ETE) with IC_50_ values ranging from 1.40‐4.18 μM. All compounds, except **1 d**, did not directly inhibit 5‐LOX in cell‐free assay, excluding the interference with this enzyme as reason for suppression of 5‐LOX product formation in intact cells. It is worth to note that this obtained biological profile was also detected for the well‐known FLAP inhibitor MK886 and for the novel FLAP inhibitor diflapolin. Moreover, the result from the proximity ligation assay suggests a direct interference of **1 d** with FLAP. Structure‐activity investigation indicated the key structural features responsible for the binding towards LTC_4_S and FLAP: the 1‐fluoro‐2,4‐dinitro‐phenyl moiety interacts with polar residues such as arginine, histidine, lysine, and also gives π‐π interactions. The rings B and C are involved in van der Waals and π‐π contacts with the macromolecule counterparts, mainly facing non‐polar residues. It is noteworthy that most of these structural features agree with observed ligand binding properties towards mPGES‐1. For mPGES‐1 we did not observe any π‐π interactions by ring A, and the fluorine atom could participate with a weak H‐bond (averaged distance from Arg52 is 3.15 Å). Overall, our analysis suggested that fluorine is not necessary for binding against all investigated targets. Thus, considering the electrophilic feature provided to the compounds by fluorine, and the consequent potential toxicity, it can be removed or eventually substituted by other H‐bond acceptors/donors in future drug design. Docked poses into mPGES‐1 catalytic cavity, showed both rings B and C interacting with hydrophobic transmembrane helixes. The nitro group at C‐2 position addresses LTC_4_S and mPGES‐1 recognition, whereas the same group at C‐4 is fundamental for FLAP binding. The nitro group at C‐4 position further contributes to the binding of FLAP and mPGES‐1 by π‐cation interaction with Phe123 and H‐bond with GSH into catalytic site, respectively. Differently, for LTC_4_S intermolecular recognition, the nitro group at C‐4 looks solvent exposed. Furthermore, unlike FLAP and mPGES‐1, the ligand length greatly affects its appropriate accommodation into the enzymatic pocket of LTC_4_S. In the para‐substituted B ring, a linker endowed with a hydrogen bond acceptor, directly bound to ring C, could contribute to affinity *vs*. macromolecule. It is worth of note that inserting the ring C at the ortho‐position of ring B is crucial to obtain the highest affinity and inhibitory activity against investigated MAPEG enzymes.

Moreover, the *ortho*‐ positioning of ring C on central cycle B can also lead to the binding towards 5‐LOX. Indeed, this position mainly favors a proper accommodation into the binding pocket and the π‐π interactions with His367 and His372 of 5‐LOX. Finally, both nitro groups are engaged in hydrogen bonds giving a fundamental affinity contribution, while the fluorine atom is not involved in any intermolecular interaction, suggesting its possible removal from the ligand structure.

These findings could pave the way for the design of multiple binders of MAPEG members. In the context of the interference of PGE_2_/LT biosynthesis, dual inhibitors are generally adopted. Interestingly, the identified 2,4‐dinitro‐biphenyl scaffold could be used to design ligands targeting more than two proteins, increasing the possibility to develop drugs for more efficient and safer anti‐inflammatory and anti‐cancer therapy.

## Experimental Section

**Molecular docking**. The ligand structures were constructed by Build Panel of Maestro (version 11) and their geometries optimized by using: the Polak‐Ribier conjugate gradient algorithm (maximum derivative less than 0.001 kcal/mol), OPLS3 force field,^[26]^ GB/SA (generalized Born/surface area)^[27]^ solvent model for H_2_O. As protein models the X‐ray structures of LTC_4_S (PDB ID: 2UUH),^[16]^ FLAP (PDB ID: 6VGC)^[17]^ and 5‐LOX (PDB ID: 6N2W)^[28]^ were employed and processed by Protein Preparation Wizard:[^29^, ^30^] checking for residue alternate positions, missing side chains and loops; bond order assignment, all hydrogen addition; the sidechain charges were given accounting for their p*K*_a_ at pH 7.0. The H‐bond network was refined by the optimize option and water molecules were removed. Molecular docking was performed by Glide software.[^31^, ^32^] Theoretical parameters were validated by docking three crystallized ligands (Figures S1‐S3).[^33^, ^34^, ^35^] An inner and outer receptor grid boxes of 10 Å and 22 Å, respectively, was used for LTC_4_S and centered on x, y, and z coordinates between chains A and C: 15.82, 7.43, −19.14. For FLAP, a sized grid of 10 Å (inner) and 16 Å (outer), was centered between chains A and C at: 65.91 (x), 83.56 (y), 32.18 (z). For 5‐LOX, the inner and outer boxes were respectively set up at 10 Å and 16 Å. The Standard Precision (SP) was employed, applying default parameters and generating one pose per ligand. The generated conformations from SP calculations were used as input geometries for three Extra Precision (XP) Glide mode rounds of predictions. The enhanced sampling mode was applied, maintaining 10000 poses/ligand for the initial step of docking, selecting 1000 poses per ligand for energy minimization. For each ligand, 1000 maximum output structures were kept, by using 0.15 as partial charge cut‐off and 0.8 as the scaling factor for van der Waals radii. A post‐docking optimization was made on docked conformations, considering 100 maximum number of poses, applying 0.5 kcal/mol as a cut‐off for rejecting obtained minimized poses. The following energy contributions were accounted: aromatic H‐ and halogen bonds (as donor and acceptor); reward of intramolecular H‐bonds; Epik state penalty. Predicted docking scores were normalized as previously described^[36]^ through the following equation: V=V_**0**_/V_**R**_, where V_**0**_ is the docking score (kcal/mol) of each ligand, and V_**R**_ is the average value (kcal/mol) of decoys. We used the online DUD−E (Directory of Useful Decoys, Enhanced) version to generate the decoys.^[37]^ Maestro (version 11) was employed for docking outcome analysis and for figure generation.

**Molecular dynamics**. The docked complexes of **1 d** bound to LTC_4_S, FLAP and 5‐LOX were used as starting models for molecular dynamics simulations and prepared by System Builder^[38]^ in Desmond,[^39^, ^40^] by using: an orthorhombic box for LTC_4_S and FLAP, and a cubic box for 5‐LOX with a 10 Å buffer distance, OPLS3 force field,^[25]^ the TIP3P^[41]^ solvation model, Na^+^ and Cl^−^ ions for electroneutrality, along with a NaCl solution (0.15 M). The LTC_4_S and FLAP were positioned into the POPC membrane through the orientation provided on the OPM (Orientations of Proteins in Membranes) database.^[42]^ The so built systems were firstly minimized by LBFGS method, 2000 iterations, convergence threshold of 50.0 kcal/mol/Å; with restrained solute heavy atoms (50 kcal/mol) and then without any constrains. The minimized molecular system were further equilibrated as follows: 0.3 ns of NVT simulation at 310 K, with restrained solute heavy atoms (50 kcal/mol); 1 ns of NPT simulation (310 K) with restrained solute heavy atoms (10 kcal/mol) and H_2_O barrier; 0.5 ns of NPT simulation (310 K) of solvent and lipids with restrained solute heavy atoms (10 kcal/mol); 3 ns of NPT simulation (310 K) with restrained solute heavy atoms (10 kcal/mol); 0.5 ns of NPT simulation (310 K) with restrained Cα protein atoms (2 kcal/mol); unrestrained 5 ns of NPT simulation (310 K). The **1 d**–5‐LOX complex was firstly optimized by the LBFGS methodology using default parameters and then underwent to the following relaxation protocol: (1) restrained solute heavy atom NVT simulation (2 ns, 10 K, small time steps); (2) restrained solute heavy atom NVT simulation (240 ps, 10 K with Berendsen thermostat, fast temperature relaxation constant) 1 ps of velocity resampling; (3) restrained solute heavy atom NPT simulation (240 ps, 10 K) with Berendsen thermostat and Berendsen barostat (1 atm), fast temperature relaxation constant, slow pressure relaxation constant, velocity resampling of 1 ps; (4) restrained solute heavy atom NPT ensemble simulation (240 ps) through Berendsen barostat (1 atm) and Berendsen thermostat (310 K), fast temperature relaxation constant, slow pressure relaxation constant, velocity resampling of 1 ps; (5) 480 ps NPT simulation employing Berendsen thermostat (310 K) and Berendsen barostat (1 atm), normal pressure relaxation constant and fast temperature relaxation constant. Unrestrained molecular dynamics of 100 ns (310 K) with NPT (1.01 bar) ensemble class were run, through 1.2 ps of recording time and 2.0 fs of integration time step. Each equilibration phase of three systems was evaluated by the Simulation Quality Analysis tool of Desmond, examining pressure, volume, temperature, total and potential energies.

**Determination of LTC_4_ synthase activity**. HEK293 cells stably expressing human recombinant LTC_4_S (HEK_LTC_4_S) were used to generate microsomes containing LTC_4_S. Briefly, HEK_LTC_4_S cells were frozen in liquid nitrogen and sonicated 3×20 s at 4 °C in homogenization buffer (0.1 M potassium phosphate buffer, pH 7.4, 1 mM phenylmethanesulfonylfluoride, 60 μg/mL soybean trypsin inhibitor, 1 μg/mL leupeptin, 2.5 mM glutathione, and 250 mM sucrose). Lysates were sequentially centrifuged at 10,000×g, 10 min at 4 °C and 174,000×g for 1 h at 4 °C. The microsomal fraction (2.5 μg) was resuspended in assay buffer (0.1 M potassium buffer pH 7.4, plus 5 mM glutathione) and preincubated with the test compound or vehicle (0.1 % DMSO) for 10 min at 4 °C. The reactions were started by adding 1 μM LTA_4_‐methyl ester (Cayman, Ann Arbor, MI) and stopped after 10 min incubation at 4 °C by 1 vol. ice‐cold methanol. Acidified PBS and LTC_4_‐methyl ester‐d_5_ as internal standard were added prior solid phase extraction, and LTC_4_‐methyl ester formation was analyzed by UPLC‐MS/MS as described.^[43]^ UPLC‐MS/MS was carried out by Acquity UPLC BEH C18 column (1.7 μm, 2.1×50 mm, waters) and QTRAP 5500 Mass spectrometer (AB Sciex). LTC_4_‐methyl ester and LTC_4_‐d_5_ methyl ester (standard) were detected by multiple reaction monitoring in the negative ion mode by a method described previously.^[44]^

**Blood cell isolation and cultivation of cell lines**. Human neutrophils were freshly isolated from peripheral blood obtained at the Institute for Transfusion Medicine of the University Hospital Jena (Germany) as described.^[45]^ Briefly, human peripheral blood was collected in heparinized tubes (16 I. E. heparin/mL blood) by venipuncture from fasted (12 h) adult healthy volunteers, with consent, and leukocyte concentrates were prepared by centrifugation (4000×g, 20 min, 20 °C). The subjects had no apparent inflammatory conditions and had not taken anti‐inflammatory drugs for at least ten days prior to blood collection. Neutrophils were immediately isolated by dextran sedimentation and centrifugation on Nycoprep cushions (PAA Laboratories, Linz, Austria) and hypotonic lysis of erythrocytes was performed under hypotonic conditions. Neutrophils were finally resuspended in PBS pH 7.4 containing 1 mg/mL glucose and 1 mM CaCl_2_ (purity >96‐97 %).

**5‐LOX activity assay**. Neutrophils (5×10^6^ cells mL^−1^) in PBS pH 7.4 plus 1 mM CaCl_2_ containing 1 mg mL^−1^ glucose were pre‐incubated with tested compounds (1 μL in DMSO; final DMSO concentration: 0.1 %) for 10 min and then treated with 2.5 μM Ca^2+^ ionophore A23187. After 10 min at 37 °C the reaction was stopped on ice by addition of 1 mL of methanol. 30 μL 1 N HCl and 500 μL PBS, and 200 ng PGB1 were added and the samples were subjected to solid phase extraction on C18‐columns (100 mg, UCT, Bristol, PA, USA). 5‐LOX products (LTB_4_, all‐trans isomers of LTB_4_, and 5‐hydro(pero)xy‐6,8,11,14‐ eicosatetraenoic acid (5‐H(p)ETE) were analyzed by RP‐HPLC and quantities calculated referencing to the internal standard PGB_1_.

**Human recombinant 5‐LOX activity assay**. Human recombinant 5‐LOX was expressed in *Escherichia coli* (*E. coli* BL21 (DE3)) cells and partially purified by affinity chromatography using an ATP‐agarose column as described.^[22]^ Semi‐purified 5‐LOX (specific activity: 1.6 ± 0.2 μg 5‐LOX products per μg protein) was diluted in PBS containing EDTA (1 mM) and ATP (1 mM) to a concentration of 0.5 μg mL^−1^ and immediately pre‐incubated with the test compounds (1 μL in DMSO; final DMSO concentration: 0.1 %) for 10 min at 4 °C. Samples were pre‐warmed for 30 s at 37 °C, and 5‐LOX product formation was initiated adding 2 mM CaCl_2_ and 20 μM arachidonic acid. The reaction was stopped after 10 min at 37 °C adding 1 mL ice‐cold methanol. Formed 5‐LOX metabolites (all‐trans isomers of LTB_4_ and 5‐H(p)ETE) were extracted and an aliquot of 50 μL analyzed by reversed phase‐HPLC (RP‐HPLC) as described.^[23]^ Data were normalized to the vehicle control to avoid variations independent of test compounds.

**Reversed phase liquid chromatography and mass spectrometry**. LTC_4_S metabolites were separated on an Acquity ultra performance liquid chromatography (UPLC) BEH C18 column (1.7 μm, 2.1×50 mm, Waters, Milford, MA) using an Acquity^TM^ UPLC system (Waters, Milford, MA, USA) as previously described.^[42]^ The chromatography system was coupled to a QTRAP 5500 Mass Spectrometer (AB Sciex, Darmstadt, Germany) equipped with an electrospray ionization source. LTC_4_S metabolites were quantified by multiple reaction monitoring in the negative using a previously reported method with a lower limit of detection of 150 to 600 pg mL^−1^ and linear quantification range up to 200 ng mL^−1^.^[22]^ Automatic peak integration was performed with ANALYST 1.6 software (Sciex, Darmstadt, Germany) using IntelliQuan default settings. Data were normalized on the internal standard PGB_1_ and are given as relative intensities.

**Cell culture**. The human embryonic kidney cell line HEK293 was cultured as monolayer at 37 °C and 5 % CO_2_ in DMEM High glucose containing 10 % heat‐inactivated fetal calf serum (FCS), 100 U/mL penicillin and 100 μg/mL streptomycin. HEK293 cells stably expressing human recombinant 5‐LOX and FLAP were selected by 400 μg/mL geneticin and 200 μg/mL hygromycin B, respectively, as described before.^[21]^

**Analysis of 5‐LOX/FLAP interaction by in situ proximity ligation assay**. The manufacturer's protocol^[46]^ for proximity ligation assay was used to detect in situ interaction of 5‐LOX with FLAP in HEK293 cells stably expressing both proteins as reported elsewhere.^[24]^ Cells were pretreated with test compounds for 10 min at 37 °C, then treated with 2.5 μM Ca^2+^‐ionophore A23187 for another 10 min, fixed, and incubated with primary antibody overnight as described before.^[24]^ Samples were then incubated for 1 h (37 °C) with species‐specific secondary antibodies conjugated with oligonucleotides (PLA probe anti‐mouse MINUS and anti‐rabbit PLUS). The antibody‐bound oligonucleotides formed a DNA circle by addition of ligase (30 min, 37 °C). Rolling‐circle‐amplification of generated DNA circle and hybridization of fluorescently labeled oligonucleotides were performed for 100 min at 37 °C. Nuclear DNA was stained with DAPI. Protein‐protein‐interactions occurred as magenta fluorescent spots analyzed with an Axiovert 200 M microscope (Carl Zeiss) and a Plan Neofluar×40/1.3 Oil (DIC III) objective (Carl Zeiss).

**mPGES‐1 washout assay**. Microsomes derived from A549 cells expressing mPGES‐1 were incubated with low (1 μM) or high concentrations (10 μM) of compound **1 d**. After 15 min an aliquot of the sample containing 10 μM **1 d** was diluted 10‐fold with buffer to reach a final concentration of 1 μM. After another 5 min the substrate PGH_2_ (20 μM) was added. The reaction was stopped after 60 s to determine PGE_2_ formation. Washout data are presented as means of residual activity ± SD in % of vehicle treated control for *n*=3 independent experiments. Outliers were determined using Grubb's test (GraphPad Software, La Jolla, CA). Samples were not blinded.

**Statistical analysis**. Data are expressed as mean ± S.E.M. of single determinations performed in three or four independent experiments at different days. IC_50_ values were graphically calculated from averaged measurements at 4‐5 different concentrations of the compounds using GRAPHPAD PRISM 4.0 software (San Diego, CA, USA). Statistical evaluation of the data was performed by one‐way ANOVA followed by Tukey‐Kramer post‐hoc test for multiple comparisons. A p value <0.05 (*) was considered significant.

## Conflict of interest

The authors declare no conflict of interest.

1

## Supporting information

As a service to our authors and readers, this journal provides supporting information supplied by the authors. Such materials are peer reviewed and may be re‐organized for online delivery, but are not copy‐edited or typeset. Technical support issues arising from supporting information (other than missing files) should be addressed to the authors.

Supporting Information

## Data Availability

Research data are not shared.
