# Supplementary material for: Identification of 2,4‐Dinitro‐Biphenyl‐Based Compounds as MAPEG Inhibitors
Source: ChemMedChem. 2022 Oct 11;17(22):e202200327. doi: 10.1002/cmdc.202200327 (PMC9827972; doi:10.1002/cmdc.202200327)
Supplement: Supplementary file 1 — Supporting Information [file CMDC-17-0-s001.pdf]

ChemMedChem

Supporting Information

Identification of 2,4-Dinitro-Biphenyl-Based Compounds as MAPEG Inhibitors

Simone Di Micco, Stefania Terracciano, Martina Pierri, Vincenza Cantone, Stefanie Liening, Stefanie König, Ulrike Garscha, Robert Klaus Hofstetter, Andreas Koeberle, Oliver Werz, Ines Bruno, and Giuseppe Bifulco*

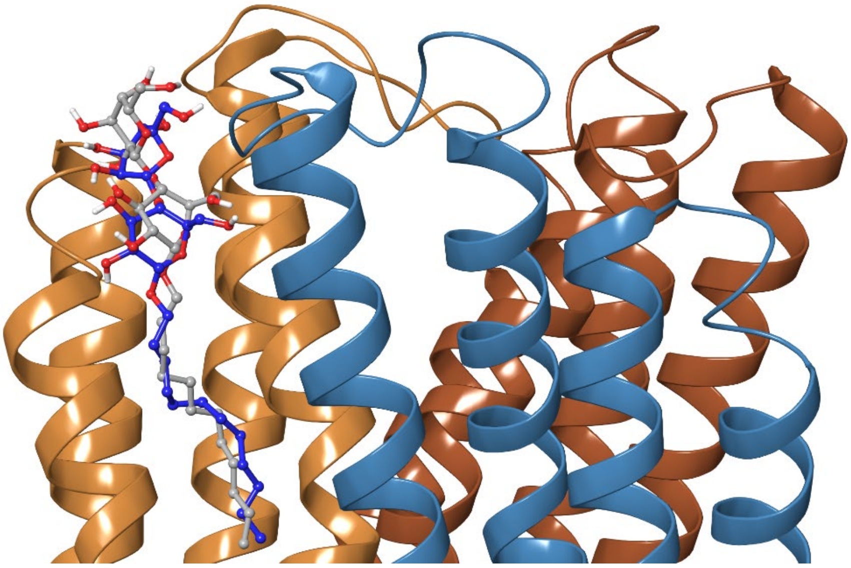

Figure S1. Superimposition (1.49 Å) of co-crystallized (green carbon, PDB ID: 5bqg) and docked pose (gold carbon) structures of dodecyl-alpha-d-maltoside. This molecule was used as tentative substrate model, due to the absence of co-crystallized ligand. The protein is depicted by ribbons (chain A, light brown; chain B, brown; chain C, azure). The ligand is represented by sticks and balls (C, as for the sticks; polar H, white; N, dark blue; O, red).

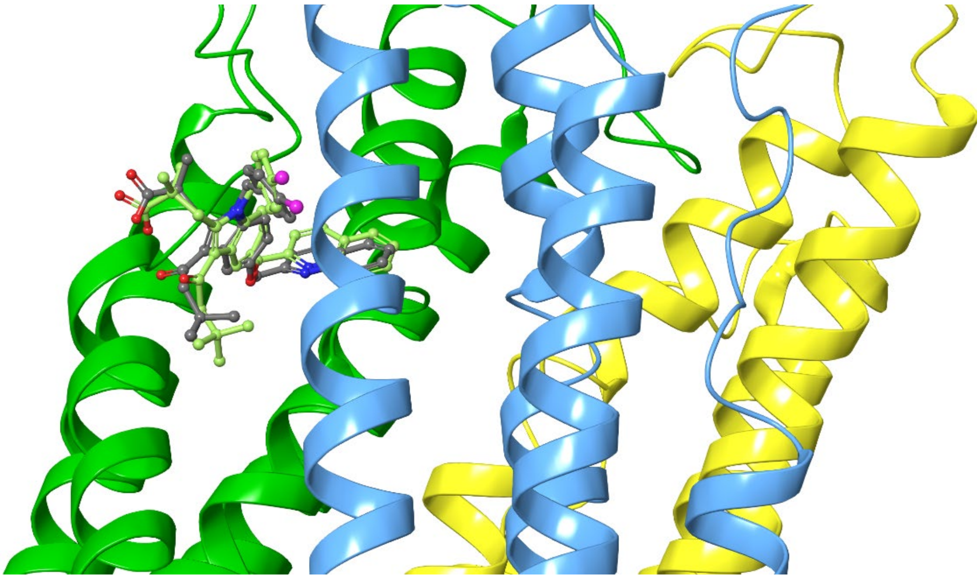

Figure S2. Superimposition (1.01 Å) of co-crystallized (grey carbon, PDB ID: 2Q7R) and docked pose (lime carbon) structures of MK-591. The protein is depicted by ribbons (chain A, green; chain B, faded azure; chain C, yellow). The ligand is represented by sticks and balls (C, as for the sticks; polar H, white; N, dark blue; O, red, I, magenta).

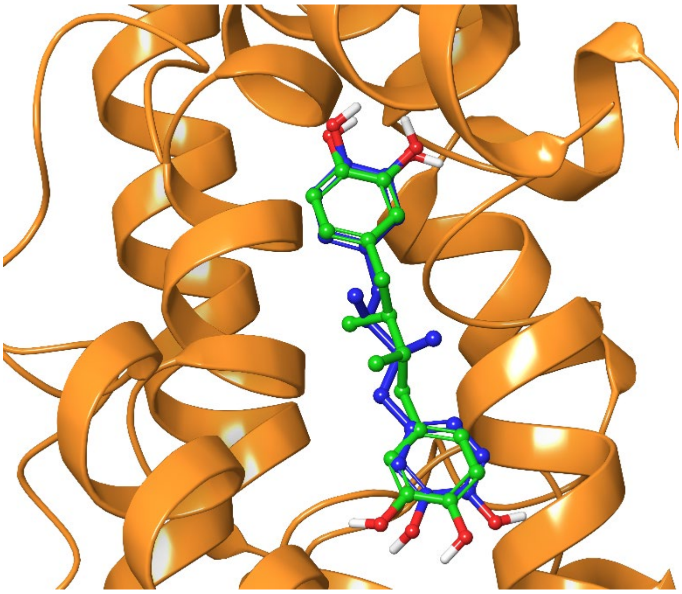

Figure S3. Superimposition (1.29 Å) of co-crystallized (green carbon, PDB ID: 6N2W) and docked pose (blue) structures of NDGA. The protein is depicted by orang ribbons. The ligand is represented by sticks and balls (C, as for the sticks; polar H, white; O, red,).
